# Alois Alzheimer: A Hundred Years after the Discovery of the Eponymous Disorder

**Published:** 2006-06

**Authors:** Antonio Tagarelli, Anna Piro, Giuseppe Tagarelli, Paolo Lagonia, Aldo Quattrone

**Affiliations:** *Istituto di Scienze Neurologiche - CNR, Mangone (Cosenza), Italy*

**Keywords:** alzheimers disease, neurology, centennial, senile dementia, biography, alois Alzheimer

## Abstract

The familiar term “Alzheimer’s disease” was coined by Emil Kraepelin to honour his pupil, Alois Alzheimer. However, little is known about the life of the man after whom this important and well-known disease was termed. On the centennial of the discovery of Alzheimer’s disease, it is appropriate to report some aspects of the life and scientific work of Alois Alzheimer. The authors contacted all the libraries of the Universities where Alzheimer studied and/or worked to receive any original material regarding Alois Alzheimer. This review is based for a most part on an original biography written by Konrad and Urlike Maurer after the interviews to Alzheimer’s nieces, Hildegard Koeppen, Ilse Lieblein, Bärbel Lippert, Karin Weiβ, and his nephew, Rupert Finsterwalder. The authors obtained this biography from the Central Library of Medicine in Koeln.

## ALZHEIMER’S LIFE

The ancestors of Alois Alzheimer (originally “Alsheimer”) were devout Catholics from Spessart, and Alois remained sentimental about this region. His great grandfather, Michael Alzheimer, was born in Rengersbrunn in 1757. He married Margarethe Günter on 7 February 1792, and their son Johann, Alois’ grandfather, was born in 1797. Johann, who became a teacher, was compelled to remove to Cassel, a town in the north-west of Low Franconia. Karl Gorge, the first son of Johann, was born on 6 October 1827. Their second son, Edward, was born on 22 March 1830. Both sons became esteemed member of society: Karl Gorge as a well-known prelate and Edward as a notary. Edward, who married Eva Maria Sabina Busch in 1861 in Würzburg and later moved to Marktbreit on the Main in Low Franconia, was the first notary to work at the royal provincial law court. Eva Maria Sabina died on 31 July 1862 at 336 Würzburger Street, a house that still stands. Edward married Therese, Eva Maria Sabina’s sister, who lived nearby at 273 Würzburger Street. This house is peculiar in that it has a central tubular space in which a birch was planted. The birch is still alive and the house was proclaimed a museum on 19 December 1995. Alois Alzheimer was born in this house on 14 June 1864. He was the second son of Edward and Therese, and was a healthy infant. He attended the catholic School of Marktbreit from 1870 to 1874, but his father believed that this city did not offer sufficient cultural depth and so sent him to Aschaffenburg when he was ten years old. Alois attended the high school there and was educated by teachers better than most in those times. In 1878, his family also moved to Aschaffenburg. During high school, Alois was attracted to natural science and concentrated on this area. In 1883, Alois obtained the general certificate of education. Faced with the choice of tertiary courses in either natural science or medicine, he chose the latter. At this time, Berlin was considered the centre of excellence in medicine and was home to scientists such as Rudolf Virchow, the cellular pathologist, and Robert Koch, who discovered the mycobacterium responsible for tuberculosis and vibrio cholerae. Inspired by these scientists, Alois relocated to Berlin and was enrolled at the Royal Friedrich Wilhelm University of Berlin from 1883 to 1884. He attended anatomy lectures and dissections under the supervision of Wilhelm von Waldeyer and studied zoology under Marteus, experimental inorganic chemistry under Adolph Pinner, and botany under August Wilhelm Eichler and Reiner Westermeier. Importantly, he attended Carl Westphal’s psychiatry lectures. Westphal’s emphasis on the role of cerebral disorders in psychosis had a formative influence on Alzheimer’s way of thinking. Alzheimer was impressed by the English principle of non-restraint that was advocated by John Conolly; this entailed treatment for mental patients without constrictive methods. In the summer of 1884, Alzheimer attended his second six-month period at Würzburg, where the left side of his face was disfigured in a fencing duel. From this time, only the right side of his face is shown in photographs. During the winter of 1884 – 1885, Alois attended the physiology lectures of Adolf Flick, who formulated the laws of microscopic physics and the mathematics of diffusion, and the microscopy lectures of Albert von Kölliker. Alzheimer worked with Alfonso Corti, who discovered Corti’s gland, and with Franz von Leydig, who discovered Leydig cells. During the winter of 1885-1886, he attended Hubert Grashey’s lectures on general pathology and legal psychiatry. The next winter he went to the Eberhard-Karl University of Tubingen where he attended lectures in specialized pathology and treatment, clinical medicine, clinical surgery, and gynaecology and obstetrics. Alois returned to Würzburg the following summer to write his graduation thesis titled, “On earwax glands”. The chairmen of his examination committee were von Kölliker and Stöhr. In Alzheimer’s words, *“the earwax gland is formed by the growth of the ending of the external root of the hair … the wax flows down into the follicle in newborns … and in adults it flows into the free areas of the skin”.* Alzheimer’s prodigious capacity as a researcher and pathologist is reflected in the tables in his graduation thesis. His analytical and descriptive talents contributed to the use of microscopy in the study of psychiatry. Two of his distinctive characteristics are that he was a good microscopist and that he placed importance on dialogue with patients. On 12 May 1888, Alzheimer passed the graduate examination and on 4 July was granted a doctors degree to practice medicine in the territory of the German Kingdom by the Royal Bavarian Statal Minister of the Church and School. On 19 December 1888, Alois replaced Emil Sioli as the assistant to Heinrich Hoffmann, director of the Institute for Lunatics and Epileptics in Frankfurt/Main. He was appalled at the inadequate sanitary measures and living conditions at the Institute, as he and Sioli espoused non-restrictive treatment of patients. Franz Nissl, who also worked at the institute, had an important influence on Alzheimer. Nissl developed Nissl’s stain and discovered Nissl’s lumps (small granules within the neurons). Sioli, Alzheimer and Nissl changed the institute into a proper psychiatric clinic and making it a caring facility. They used a non-restrictive policy characterized by bath therapy and dialogue between physician and patient. In Alzheimer’s words, *“all restrictive methods were abandoned … [using methods such as] introducing an intensive medical service, introducing treatment next to the bed of the patient and into the day room, avoiding the isolation room, introducing permanent baths. [Sioli’s Institute] is one of the most progressive institutes”.* A very important element for the three physicians was research into the organic causes of mental diseases using histological preparations made during autopsy. In April 1894, Alois married Cecilie Simonette Nathalie Wallerstein in the Civil Status Office of Frankfurt. When Cecilie converted to Catholicism on 14 February 1895, their marriage ceremony was conducted in the Franciscan Church in Frankfurt. A daughter, Gertrude, was born on 10 March 1895 and Nissl was named as her godfather. On 21 July 1896, Alzheimer was appointed head of the mental hospital in Frankfurt. Their son, Hans, was born two days after his appointment. The closing years of the nineteenth century were very profitable for Alzheimer but the year 1901 was to be pivotal for his future: his status as a professional was firmly established and the Institute was expanded, but his wife died. During the first years of the twentieth century, Alois abandoned his academic aspirations for a more applied role in which he could link theoretical and practical science. He moved to Heidelberg at the invitation of Emil Kraepelin. When Kraepelin took up a chair in psychiatry in Munich in June 1903, he asked Alois to accompany him. In Kraepelin’s words, *“Before his establishment as university lecturer in Heidelberg it was my appointment in Munich where he went together with me”.* On 9 November 1904, Kraepelin and Alzheimer opened their psychiatric clinic. The institute was relatively modern in respect of therapy and hygiene. There were no isolation rooms, and all patients were treated using baths. According to their circumstances, patients were admitted for days, weeks or months. About 2,000 patients were admitted each year. On 16 November 1904 Alzheimer was appointed a lecturer in the Faculty of Medicine at Ludwig-Maximilian University. He then applied for *venia legendi*, leave to teach in psychiatry, and was appointed a private teacher. Alzheimer was very devoted to his work, but also spent much time in educating and instilling a love of nature in his sons. His son, Hans, wrote, *“it is very important to my father that we possess animals and love them”.* Their house, where Alzheimer’s niece presently resides, borders on Weβlinger Lake and was thus very suitable for such pursuits. Despite his almost military gait, Alois had a quiet nature and a great sense of humour. He loved good cigars, and had a habit of leaving-and forgetting-his partially smoked cigars on students’ desks so that by evening the laboratory was filled with cigar smoke. On 22 February 1909, Alzheimer resigned from office to devote himself to scientific research, and on 30 December 1909 was granted a teacher’s degree in the Court of Bavaria by Prince Luitpold. Three years later, he was appointed as ordinary professor of psychiatry at the neurologic and psychiatric clinic of the Friedrich-Wilhelm University in Breslau. This was important for his scientific career: he competed with Eugen Bleuer, researcher of cerebral anatomy and pathology; Paul Schröeder, head of the clinic in Breslau; Oswald Bumke, head of the clinic in Freiburg. The minutes of the appointments committee describe him as,*“… a great authority for the studies of the histology and pathology of the brain, and he has successfully studied organic psychosis, paralysis, cerebrallues, arteriosclerosis and epilepsy … he is always interested to show the comparison between the clinic and the clinical diagnosis … his studies considered landmarks from a clinical point of view … he is a good teacher and a good physician … the faculty is persuaded that he would accomplish his task in an optimum way”.* On 10 July 1912, Emperor Wilhelm II personally signed the appointment. Alzheimer went to Breslau but became severely ill. Kraepelin described his affliction as, *“infective angina associated with nephritis and inflammation of the articulations [contracted] during travel to the new residence”.* He continued to work despite his illness. In the summer of 1913, Alzheimer taught courses in psychiatry and its relationship to the anatomy and pathology of the brain, and histology and histologic pathology of the nervous system at the University of Breslau. Ludwig Mann, Ottfried Förster, and Georg Sterz were coworkers of Alzheimer. Stertz was to become Alzheimer’s son in law. In February 1913, Alzheimer was hospitalized in a private clinic. His spirits remained high, but by the end of November, he developed severe renal and respiratory failure and died on 19 December 1915. Many obituaries were written. Of particular note are the words of Clemens Neisser at the Congress of the Oriental German Association for Psychiatry in Breslau on 9 December 1916: *“… in the Congress on 7 December 1912 in which he agreed to the Association, with his conference paper on ‘About some previously unknown clinical cases’, he opened a common element of stuffy on the paralysis for the clinic and provincial Institute. He allowed us to enter into his personal fields of studies, and so, we hope, in a common and scientific union among scientists and a promotion of our own work”* (1-4).

## SCIENTIFIC ACTIVITIES

It is important to stress that the scientific thinking of Alois Alzheimer, from beginning, influenced by that of Heinrich Hoffmann. In 1851, Hoffmann was director of the Institute for Lunatics and Epileptics in Frankfurt and in 1859, he wrote “Observations and experiences about psychical disorders and about epilepsy in the Institute of Frankfurt am Main from 1851 to 1858”. Hoffmann belonged to the school of “somatics”, who considered all psychic diseases to be effects of organic diseases, as did Alzheimer. This group was in opposition to the “psychics”, who considered the psyche to be the cause of mental diseases. Hoffmann followed the teaching of Wilhelm Griesinger, a psychiatrist from Berlin who initiated the scientific era of psychiatry with his treatise on “Pathology and therapy of physical disorder”. The essence of Griesinger’s teaching is that *“psychical disorders are brain disorders”.* Moreover, Heinrich Hoffmann based his principles on non-restrictive or free treatment, which became the basis of Alzheimer’s work. The first publication by Alzheimer and Nissl describes a patient with progressive general and muscle weakness due to degeneration of the grey matter of the spinal cord and diffuse damage to the neurons of the cortex. Alzheimer’s diagnosis of progressive spinal muscular atrophy was confirmed by an autopsy. Alzheimer had three main tenets: non-restrictive treatment, dialogue therapy, and research into the organic causes for the psychical diseases. At beginning of his scientific activity, he made over 200 histological preparations from patients with syphilis. He worked in the pathological anatomy section with Carl Weigert, the inventor of aniline staining, and Ludwig Edinger, who discovered pathways for pain and temperature sensibility, who both assisted him. Alzheimer used Nissl’s stain for his preparations and advised *“… the use of this method everywhere is urgent, not only in those cases that are characterized by a loss of fibres, but also to show the pathological changes of the neurons”.* In 1892, Alzheimer wrote a detailed report on the positive effects of treating mental patients using baths and presented it in Karlsruhe to the Congress of Psychiatrics of South-West Germany. He described arterisclerosis of the brain in 12 syphilis-free patients at the meeting of the Association of German Psychiatrics in Dresda on 21 and 22 September 1894. In this communication, he emphasized that the clinical signs of arteriosclerotic atrophy differ from those of other diseases. A characteristic feature of this disease is a hearth’s image of the arteriosclerotic degeneration respect to diffuse paralytic changes. Alzheimer anticipated dementia that is caused by multiple infarcts a century before its discovery. On the causes of the arteriosclerosis, Alzheimer wrote, *“I can exclude trauma, diabetes, nephritis, or a non-compensated cardiac insufficiency … I believe that my patients are near to senile dementia and someone considers them as a progressive paralysis. I can separate them clearly by praecox senile dementia”.* This is a very important affirmation because the difference between pre-senile and senile dementia played a key role in Alzheimer’s diagnosis of Auguste D. seven years later. At the 66^th^ Congress of Psychiatrics in Vienna, Alzheimer the age at which progressive paralysis develops: *“I studied three young girls. I wish to describe one case as an example. I studied an illegitimate daughter … when 21 years old, was hospitalized due to a reduced visual capacity. This was diagnosed as atrophy of the visual nerve, headache, backache, and loss of peculiar reflexes; when the patient was 26 years old, she was hospitalized in our institute because she was very excited. She exhibited lingual fibrillar twitching, twitching of the face muscles, dementia, paralytic signs, temporary excitation … our colleagues, who assigned us the patients, had difficulty with the diagnosis as each had a different opinion … but nobody diagnosed paralysis”.* Alzheimer’s faith in microscopy and pathological anatomy is evidenced by his statement, *“I am sad to affirm that only autopsy will provide certainty”.* He published a report on these three cases in 1896, showing that the paralysis begins between 13 and 16 years of age and lasts for five years, which is longer than is the case with adult paralysis. In contrast to adult cases with this disease, in which males become more clinically ill than females, in juveniles, both sexes fall ill and there is no clinical difference between them. In 1896, Alois Alzheimer published an interesting article, “A born felon” in the Archive of Psychiatry and Nervous Diseases. He describes Oskar M., who was admitted to the mental hospital on 16 March 1894. Alzheimer measured the cranium and documented a marked asymmetry of the face and cranial bones, which was diagnosed as an inherited mental degenerative disease according to Benedict Morel. From Alzheimer’s words, *“We believe that the disorder of Oskar M. is a hereditary degenerative disorder. The more evident symptom is a sexual perversity showing a typical fetishism. Moreover, a great obsession united to signs of a forced action are present … Moreover, Oskar M. shows character anomalies which are peculiarities of those who show psychical hereditary vices”.* Alzheimer was well acquainted with the theory of Cesare Lombroso regarding the “delinquent man”. However, Lombroso believed that the stunted growth of the cranial bones was because the patient had the mental and physical traits of ancient man. In 1897, Alois published a work about psychoses resulting from nervous exhaustion entitled “Contribution to the pathological anatomy of the cerebral cortex and of the anatomical bases of some psychoses, in which he described pathological changes of neurons in the cortex, its supporting tissue, and in the connective tissue. In 1898, he published “A contribution to the pathological anatomy of epilepsy” in the Journal for Neurology and Psychiatry. Referring to the patients in his clinic, he wrote, *“There are cases of epilepsy which are said to be ‘genuine’ or ‘inbred’, and are characterized by pathological-anatomical changes. From a macroscopic perspective, it is evident that there is a wrinkled surface of the circonvolution characterized by a hunch image and a sclerosis … From a microscopic perspective, an increase of glia is evident … a great loss of fibres of the marrow and of neurons in the cerebral cortex. The degeneration extends along the all of the cerebral cortex … many reasons lead us to believe that the nervous substance is ill primarily … All the histological changes explain the presence of epilepsy”.* In 1898, Alzheimer published “New contribution on senile dementia”, a pathology in which the brain shows signs of a praecox aging in the Journal for Psychiatry and Neurology. Alzheimer had previously studied senile dementia in Frankfurt: *“Then I examined a patient showing a presenile dementia related to numerous atrophic nervous cells without any change to the ateromatous vases. Leaving nutritional disorders out of consideration, this disorder shows … a weakness of the central nervous system, which can be acquired due to hereditary vice and have praecox atrophy of gangliar cells as a consequence. Admitting this … in typical cases of senile dementia … the degenerative changes of gangliar cells can be present … naturally other research can confirm this diagnosis”.* These studies aroused Alois’ interest in senile dementia, which led to his concern with Auguste D. In 1901, Alzheimer began to study a subcortical arteriosclerotic encephalopathy, later known as Binswanger’s disease, in which *“… the patient become lame, dull-minded, physically flabby, talkative, shook”.* His scientific reputation was reinforced by his participation in various congresses. In September 1895, he presented microscopic preparations from two patients with brain degeneration to the Congress of the Association of German Psychiatrists, which was held in Hamburg. In 1896, at the Congress of German Researchers and Physicians, held in Frankfurt/Main, Alzheimer gave a talk on “The anatomical propagation of paralytic processes of degeneration”. In 1896, he talked at the Annual Congress of German Psychiatrists in Heidelberg about five patients showing arteriosclerosis. In 1898, Alzheimer talked about *delirium acutum* and mental disorders of aging at the 22nd Congress of Oriental German Psychiatrists held in Baden-Baden. In 1903, Alzheimer qualified for university teaching with his dissertation, “Histological studies about progressive paralysis”, which contained 11 tables and photographs taken by Alzheimer. This monograph was the foundation for subsequent studies on mental disorders, as it shows that pathological anatomy is an important research tool. Alzheimer then turned to the study of psychoses, dementia, schizophrenia and depressive maniacal madness. In 1904, he published “Something about the anatomical basis of idiocy” in the Central Journal for Psychiatry: *“… observing macroscopically the brain of the idiotics it is evident some peculiarity as the microencephalia and the macroencephalia … the microgiria and the macrogiria … the poroencephalia and the hydrocephalia …”.* Moreover, he wrote about amaurotic idiocy, a disorder of fat metabolism that causes psychomotor degeneration and paralysis. In 1907, Alzheimer published “Some methods to fix the cellular elements of cerebral-spinal fluid” in the Central Journal for Psychiatry in which he described the technique of lumbar injection. In the same year, he was invited make a presentation to the German Association for Psychiatry in Frankfurt because of his expertise on the etiology of various mental diseases. He discussed brain lesions, brain tumours, idiocy, encephalopathy, syphilis, alcohol poisoning and lead poisoning. Later that year, he published a detailed article titled “About indications for the artificial interruption of pregnancy in psychic patients” in the weekly Journal of Medicine in Munich and began writing a manual on the histologic pathology of psychosis with the aim of distinguishing schizophrenia from depressive maniacal madness using histological preparations. In 1909, Alzheimer wrote an article titled “Contribution about the knowledge of the pathologic neuroglia, and its relationships with the course of the decomposition of the nervous system”, which he published in 1910 in the journal Histologic and Histopathologic Studies on Cerebral Cortex. Following an article that Alzheimer published in 1911, “Is the institution of a psychiatric division within the Royal Igienic Office desiderable?”, Kraepelin founded the German Institute for Psychiatric Research in Munich, now know as the Max-Planck Institute of Psychiatry. Alzheimer never forgot his patient, Auguste D., who he studied in Frankfurt during the early years of his scientific career. When Auguste D. died on 9 April 1906, Alzheimer studied his brain to confirm his “ancient” hypothesis of senile dementia. Assisted by Gaetano Perusini and Francesco Bonfiglio, Alzheimer detected atrophy of the cerebral cortex accompanied by the loss of cells, a great excrescence of the glia of the fibres, and numerous glial rod cells. Moreover, numerous plaques throughout the cortex were associated with excrescences of the vessels. This pattern, which could hardly be described as atypical for a patient of 70 to 80 years of age, is significant because Auguste D. was only 50 years of age when he died. Alzheimer presented these results at the 37th Congress of Psychiatrists of Southern Germany in Tubingen. The Meeting was attended by prominent scientists such as Oswald Bumke, after whom the “Bumke’s syndrome” in schizophrenia was named; Otto Binswanger, after whom the “Binswanger’s disease” was named; and Hans Curschmann after whom the “Curschmann-Steinert disease” was named, but nobody attached importance to Alzheimer’s results. In 1909, Perusini published the case of Auguste D. together with three other cases in an article titled “On the psychic disorders in the late age which are peculiar from a clinic and histopathologic point of view” in Nissl and Alzheimer’s Histologic and Histopathologic Studies on the Cerebral Cortex. In the preface to his 1910 book, general Psychiatry and Clinical Psychiatry, Emil Kraepelin thanked Alzheimer: *“I express my satisfaction to my faithful co-worker, Professor Alzheimer, who allowed me to insert the results of pathologic anatomy of the Institute useful for my tables and images”.* Moreover, Kraepelin included a section titled “Senile cerebral sclerosis or Alzheimer’s disease” in chapter VII, “Senile and pre-senile madness”. On page 624, Kraepelin wrote that *“the clinical interpretation of Alzheimer’s disease is, until today, still not clear. In spite of the clarity of anatomical evidence showing a serious type of senile dementia beginning at about fifty years … in these patients it may be called ‘praecox senium’”.* The details of the disease of Auguste D. were found in 1995. The Chronicle in Munich of 4 June 1997 reports that *“For two years, Maurer, Volk and Gerbaldo searched records of patients … 12 patients called A.D. were traced … but nobody was coincident with Alzheimer’s description of Auguste D. … the real record was mistakenly collected in the year 1920”.* In 1909, Alzheimer published “Studies on the fundaments of the motorial disorders and histological changes of epilepsia”. In 1910, Alzheimer became editor of the psychiatric section of the Journal for General Neurology and Psychiatry. In the first edition, he wrote articles on “The diagnostic difficulty in Psychiatry and degeneration and regeneration of peripheral nervous vessels”. The period from 1903 to 1912 was his most fruitful. In Nissl’s words, *“Alois was very impartial in his judgements, unwilling to overstate or speculate, … zealous in doing his duty, he is not only a defender but a supporter of this research’s direction”.* In 1913, Alzheimer gave lectures and wrote treatises; he published 11 articles on dementia praecox in the Journal of General Neurology and Psychiatry and published a review of psychiatry on the occasion of the venticinquennial of his professional, Emil Sioli. In November 1914, Alzheimer gave his last lecture, on the “Mortal effect of war on the nervous system and on the psyche”, and wrote one last publication, “The war and the nerves”. The first case of Alzheimer’s disease was described by Gonzalo R. Lafora, a histopathologist from Madrid, and coworker of Ramon y Cajal (who was winner of the Nobel Prize in 1906, for expressing “the neuronal theory”. Together him, in the same year, Camillo Golgi was winner of the Nobel Prize for discovering “the black reaction”), in a manuscript titled “Uber das vorkommen amyloider korperchen im inner der ganglienzellen; ein beirtrag zum stadium der amyloiden substanz in nervensystem” published in Virchow’s Archiv. Pathol. Anat., CCV, 295-303, 1911. We wish to honour the scientific activity of Alzheimer in the words of the rector of the Friedrich-Wilhelm University of Breslau, *“The Medical Science misses … one of the best researchers … all the mad men miss a worried father … the war hospital misses a friend and a soldier”*.

## APPENDIX

We wish to report some passages from the work published by Alzheimer in 1911, “Über eigenartige krankeitsfälle des späteren alters”, and make reference to the translation, “On certain peculiar disease of old age”, which was published in History of Psychiatry, vol. 2, 1991. The article describes a detailed case presented by Alzheimer in 1906 at the meeting of South-West Germany psychiatrists. It is a classic illustration of the histology of senile dementia. From the Introduction by Hans Förstl and Raymond Levy: “… it is this paper which clearly establishes Alzheimer’s central role and which fully justifies the attachment of his name to this intriguing disease … The text should be considered as a work of reference rather than one which can easily be read from end to end …”. From Alzheimer: “…In 1906 I described a case of disease of the presenium which during life presented features different from those of recognized diseases and which a microscopic examination showed alterations of the cerebral cortex that were then unknown … various focal symptoms, mostly of an aphasic and asymbolic kind, were noticeable … no suggestion of paralytic, luetic or arteriosclerotic disease … and because senile dementia was out of the question since the patient was only 56 years of age and the clinical picture unlike that of this condition, this case not be categorized among known disorders … the microscopy of Bielschowsky stained tissue showed a clotting of fibrils which changed their staining properties and outlived the cellular disintegration … In the 8th edition of his Psychiatry Kraepelin produced a summarized account of this disease which he called Alzheimer’s disease”. The author continues to report the diagnosis of his other colleagues: “… The patches in the cortex had in the meantime observed in presbyophrenia by Fischer … and considered them as a characteristic feature of that disorder … I had myself observed and described them in dementia senilis using Nissl and Weigert Staining. I had not however realized that they corresponded to the images seen in Bielschowsky stained preparations. Perusini has pointed out that the fibrillary changes in nerve cells which I had described are also seen in severe cases of dementia senilis and Fischer has expressed the same view. The question therefore arises as to whether the cases of disease which I considered peculiar are sufficiently different clinically or histologically to be distinguished from senile dementia or whether they should be included under that rubric … Perusini felt that these cases represented a separate disease, partly for clinical and partly for histological reasons. The clinical differences were the early onset, and the presence and severity of focal symptoms which were not thought to be a feature of dementia senilis, the anatomical differences being the greater severity of the histological changes although they develop at an earlier age … we are dealing with the case of a 56 years old woman and of Perusini’s 46 year old man, in whom nobody would have made a clinical diagnosis of senile dementia. Whereas general paralysis shows similar features whether it occurs in the young or the middle aged, the symptoms in our cases are remarkably different from simple presbyophrenia … Should we ever come to the conclusion that severe senile cortical changes may occur in the forties … However, it would still not explain why these cases differ from simple senile dementia and have such severe symptoms”. Alzheimer demonstrated the difficulty of making a differential diagnosis between senile dementia and a normal aging. He studied a patient who was 54 years old and shows clinical signs of senile dementia at a relatively young age. He did not have a tumour, general paralysis, arteriosclerosis, or circulatory alterations. Alzheimer attested to the importance of autoptic microscopic examination. From Alzheimer: “… Microscopical investigation showed the cortex to be filled in varying degrees of Fischer’s plaques … They were numerous in the frontal lobe, scarce in the central gyri, present in enormous numbers in the parietal and partly also in the temporal lobes and again less numerous in the occipital lobe. In the striatum, lentiform nucleus and thalamus they were also present in abundance. Within the cerebellum they occurred abundantly … Single plaques were also visible in the grey matter … In the spinal cord I only saw a solitary one … Among the plaques in the cerebral cortex many were of an extraordinary size, such as I have never seen, even in the cornu ammonis of senile dementia … Some evidently arose from the fusion of smaller ones since they contained several central cores but others had one exceptionally big central core and an uncommonly large halo … an exceptionally severely diseased area which has been stained by Weigert’s glial impregnation combined with a modification of Mann’s staining method … we perceive an extraordinary number of dark spots of globoid, egg-shaped … In the centre of many of them, a large central body can be recognized … In the smaller plaques it mostly appear circular … In the larger plaques it is mostly circular, often with somewhat irregular edges … compared to what is observed in ordinary cases of senile dementia, we see a far larger number of fibre producing glial cells lying in the immediate vicinity of the plaque … Using the staining technique adopted one only occasionally sees an axis cylinder which enters the plaque … There is no close relationship between the foci and the blood vessels”. In this report, Alzheimer gives a reliable description of the different staining methods used. From Alzheimer: “… This demonstration of the plaques by Mann’s staining, which demonstrates them extremely clearly, has to be supplemented by other methods. It is surprising how few appear in Nissl’s cell preparation even in those parts of the cortex which are severely affected … the stain is clearly retained only by the plaque core … The Nissl stained preparation are surprising, not only because of the relative invisibility of the plaques but because of the demonstrable disturbance of the cortical architecture and the disruption of nerve cells which appear small in relation to clinical severity … Next, we see something of interest in the Herxheimer stained preparations … the plaques appear quite clearly because their cores are decisely sown with great red granules … They also gradually disappear after a few weeks in glycerol … while the scarlet stained granules … remain … In the Bielschowsky preparations something similar can be established … There are extraordinary rich accumulations of lipid granules in the ganglion cells … such a complete lipotd degeneration is seen only in senile psychoses … In Weigert’s glial preparations an enormous increase in fibroid glia is to be seen. At the moment we employ Weigert’s method … using this modification one can demonstrate the subtler glial fibres much better … In the Bielschowsky preparations, plaques are seen in the same frequency, form and order as in the Mann’s preparation … there are formations which at first sight remind one of the structure of nerve endings as we know them in muscle or receptor organs. Apart from these very beautifully developed, clearly contoured, and well structured formations, one sees more numerous ones, which are ill defined, broken up, condensed or in a state of disintegration … I still believe in their nervous origin … The fibrils show alterations, which have repeatedly been described in dementia senilis: condensation and granular decay”. We wish to report the conclusions of Alzheimer: “… However, why these cases which occur in presenile age are in general accompanied by such exceptionally severe histological alterations and why they cause such exceptionally severe symptoms cannot be answered at the moment … Anatomical research had taught us that progressive paralysis can occur until the seventies and senile disease processes as early as the forties … just as atypical localizations of the paralytic disease process can cause clinical features which differ greatly from the ordinary features of paralysis, the senile disease process can cause disorders which earlier nobody would nor could have considered as a senile disease process because of the clinical symptoms. These observations … demonstrate to us in an impressive way how difficult it is to define diseases solely with respect to their clinical features, especially in the case of those mental disorders which are caused by an organic disease process”.
